# Insights into pathologic mechanisms occurring during serious adverse events following live zoster vaccination

**DOI:** 10.1128/jvi.01816-24

**Published:** 2025-01-17

**Authors:** Peter G. E. Kennedy, Charles Grose

**Affiliations:** ^1^School of Psychology and Neuroscience, College of Medical, Veterinary & Life Sciences, Garscube Campus, University of Glasgow 223860 , Glasgow, Scotland, United Kingdom; 2 Division of Infectious Diseases, Virology Laboratory, Department of Pediatrics, University of Iowa 160412 , Iowa City, Iowa, USA; Universiteit Gent, Merelbeke, Belgium

**Keywords:** varicella-zoster virus, varicella vaccine, VZV ORF0, acute retinal necrosis, single nucleotide polymorphisms, pathogenesis

## Abstract

An effective live zoster vaccine has been widely used around the world. Although no deaths occurred in the original large clinical trial, we analyzed 10 serious adverse events, including six deaths that have subsequently occurred in four countries. The goal is to define the viral pathogenesis of these unexpected adverse events secondary to a viremia with dissemination of the vaccine virus. We also propose a new hypothesis for acute retinal necrosis that occurs post-immunization.

## INTRODUCTION

The goal of this Gem article is to re-examine aspects of varicella-zoster virus (VZV) pathogenesis, based on serious adverse events following immunization with live vaccine virus. VZV is the only human herpes virus that causes two distinguishable diseases, each with its own name: varicella (chickenpox) is the primary infection, usually occurring in childhood, following which the virus exists in latent form in neuronal ganglia until it emerges in late adulthood as the disease herpes zoster (shingles) (1). A major correlate for herpes zoster is waning cellular immunity toward VZV with advancing age. Herpes zoster may lead to an extremely painful neurological condition called post-herpetic neuralgia (2). Herpes zoster is also more frequent in younger VZV-seropositive immunosuppressed adults with decreased cell-mediated immunity, sometimes without a rash (zoster sine herpete) (3). For the above reasons, a vaccine was designed based on the same virus as contained in the live varicella vaccine to prevent both zoster and post-herpetic neuralgia. The zoster vaccine contains 14× more infectious units per vial than the varicella vaccine (4). Accordingly, a large clinical trial of a live zoster vaccine (Zostavax Merck), led by Oxman and others, showed that the incidence of both zoster and post-herpetic neuralgia was lower in immunocompetent individuals over 60 years who received the vaccine (4).

Since 2005, the live zoster vaccine has been licensed in over 55 countries, and millions of doses have been administered (5). In a 10-year review of post-marketing safety published in 2017, scientists from Merck & Company reported a fatal outcome in 0.3% of live zoster vaccine recipients and concluded that these fatalities appeared to be a temporal association and not a causal association (5). No deaths had been reported in the original clinical trials carried out mainly in U.S. military veterans in 2005 (4). The distribution of the live zoster vaccine was discontinued in the United States in 2020. However, a safety review of a nationwide live zoster vaccine campaign in Australia published in 2023 reported three deaths (6). The three deaths occurred after the administration of 1,089,966 doses of vaccine; the investigators concluded that the deaths were likely caused by inadvertent vaccination of immunocompromised people (4, 6). Because of this report, we carried out a literature search on serious adverse events following live zoster vaccination and located a total of 10 reports of viral dissemination. During this search, we did not find any other publication that had compiled and analyzed the 10 case reports. In order to define the pathologic mechanisms of these unanticipated adverse events, we will systemically review the 10 case reports. In particular, we speculate about the rare adverse event of acute retinal necrosis.

## DESCRIPTION OF INDIVIDUAL ADVERSE EVENTS IN 10 SUBJECTS

A prior analysis was undertaken of all serious adverse events that occurred in the years after children received the live varicella vaccine, in completion of a research grant protocol from the National Institutes of Health, which funded studies to advance vaccine safety (7). The methods for the literature search were described in detail in that publication (7). Because both the live varicella vaccine and the live zoster vaccine contain the same virus strain, the keywords in the earlier search also retrieved articles about serious adverse events following the administration of the live zoster vaccine. These latter reports are enumerated here and also assembled in Table 1.

**TABLE 1:** Reports of dissemination after administration of live zoster vaccine^*a*^

Case	Age	Sex	Disease	Days	Steroid/other	Antiviral/death	Reference
1	62	M	Granulomatosis	45	Y/Y	Y/Y	(8)
2	79	M	Chronic leukemia	14	N/N	Y/Y	(9)
3	71	M	Chronic leukemia	19	N/N	Y/Y	(10)
4	70	M	Rheum arthritis	30	Y/Y	Y/Y	(11)
5	>70	ND	Rheum arthritis	ND	Y/Y	ND/Y	(6)
6	>70	ND	Melanoma	ND	Y/Y	ND/Y	(6)
7	49	F	Transplantation	21	Y/Y	Y/N	(12)
8	78	F	Rheum arthritis	72	N/Y	Y/N	(13)
9	64	M	Immunocompetent	7	N/N	Y/N	(14)
10	68	F	Immunocompetent	270	N/N	Y/N	(15)

^*a*^ The viral isolates from all 10 patients were confirmed as varicella vaccine virus by a National Reference Laboratory in the United States, the United Kingdom, Canada, Mexico, or Australia. Case 3 was also reported again in the article with cases 5 and 6. Case 8 is the patient with acute retinal necrosis. The column called “Days” lists the number of days between vaccination and the adverse event. The column called “Steroid/other” indicates whether the patient received prednisone or other medications. The dosages are described in the text. The column called “Antiviral/death” indicates whether the patient received antiviral treatment and whether the patient died during the adverse event. F, female; M, male; N, no; ND, no data given; Y, yes.

Reports of six fatal and four nonfatal adverse events were found in seven different journals (Table 1). The first case was a 62-year-old man from the United States with Wegener granulomatosis (8). He was receiving prednisone (40 mg/day) and mycophenolate mofetil (1 g/day). In 2015, he was admitted to the hospital because of a vesicular rash spreading from his face to his trunk and legs. He had been given one dose of live zoster vaccine 47 days earlier. A punch biopsy of a skin vesicle was sent to the Centers for Disease Control (Atlanta, USA) and identified as varicella vaccine strain by identification of mutations in ORF62. The patient subsequently died from pneumonia and respiratory failure while on antiviral treatment. The second case was a 79-year-old man with a diagnosis of chronic lymphocytic leukemia, who lived in Scotland, UK (9). The patient had not received any cancer medications for 6 months. But he had received one live zoster vaccination during this interval; 4 weeks later, he developed a disseminated vesicular rash. In spite of hospitalization and antiviral treatment, the patient died from multi-organ failure 25 days after admission. The virus within the vesicles was identified as varicella vaccine strain by virology laboratories at the Royal Infirmary in Edinburgh and the Great Ormond Street Hospital in London, UK.

The third case was a 71-year-old Australian man with a diagnosis of chronic lymphocytic leukemia (10). The patient developed a bilateral vesicular rash nearly 3 weeks after administration of live zoster vaccine. The herpes zoster exanthem on the face was subsequently complicated by signs of viral meningoencephalitis, leading to death while on antiviral therapy. The virus was identified as varicella vaccine strain by the Victorian Infectious Diseases Reference Laboratory, Melbourne, and the Institute of Clinical Pathology and Medical Research, Sydney, Australia. The fourth case was a 70-year-old Canadian man with rheumatoid arthritis, who was being treated with a small daily dosage of prednisone (10 mg/day) together with methotrexate (2.5 mg/day) and hydroxychloroquine (200 mg/day) (11). The patient developed a vesicular rash 2 weeks after being given live zoster vaccine. The rash progressed and the patient soon developed multi-organ failure and died. The isolate from the vesicular rash was identified as a varicella vaccine strain at the National Microbiology Laboratory of Canada, Winnipeg.

The fifth and sixth fatal cases, also from Australia, were not described in detail (6). Both patients were at least 70 years old. Case number 5 had rheumatoid arthritis that was under treatment with hydroxychloroquine and prednisone (dosages not given). Case number 6 had melanoma being treated with corticosteroids and immune checkpoint inhibitors (dosages not given). Both developed fatal viral dissemination after receiving a live zoster vaccination. Samples were sent to the same laboratory as for case 3. Although this Gem article is not about medical practice, the Australian investigators state that the guidelines to avoid the administration of live zoster vaccine to immunocompromised patients may not have been followed in some cases.

The seventh case was a 49-year-old Mexican woman with a past history of kidney transplantation and diabetes (12). She was receiving azathioprine (75 mg/day), tacrolimus (8 mg/day), metformin (500 mg/day), and prednisone (5 mg/day). She developed a disseminated vesicular rash 3 weeks after receiving an injection of live zoster vaccine. Viral DNA in the blood was identified as vaccine strain by the National Institute of Medical Science and Nutrition in Mexico City. She recovered while on antiviral therapy. The eighth case was a 78-year-old British woman with rheumatoid arthritis and diabetes, who was being treated with methotrexate (7.5 mg/day), folic acid (5 mg/day), insulin (30 units/day), but no corticosteroids (13). Six weeks after receiving a live zoster vaccine, she complained of impaired vision. A white lesion was observed in her left eye, consistent with acute retinitis. A sample of vitreous fluid was positive for vaccine virus, confirmed by the Great Ormond Street Hospital virology laboratory in London. The diagnosis of acute retinal necrosis was made; the condition responded to antiviral therapy. The ninth case was a 64-year-old healthy man from the United States, who could not recall a history of childhood varicella (14). He was not taking any prescribed medications. Within a week after receiving a live zoster vaccine injection, he developed a rash that slowly disseminated over his entire body. He responded to antiviral therapy. Samples sent to Merck & Company were identified as vaccine strains. The individual was seronegative for anti-varicella IgG antibody in a blood sample taken at the onset of the rash. In retrospect, this otherwise healthy man probably was experiencing a primary varicella infection (from the zoster vaccine) and did not have varicella as a child.

The 10th case was a 68-year-old woman from the United States, who had received a live zoster vaccination in March 2012 in the upper left arm (15). In December 2012, she developed a zosteriform rash under the upper left arm that extended onto the back and chest. She recovered after treatment with oral acyclovir for 10 days. She had no history of any chronic illness nor was she taking any immunosuppressive medications. A sample from the rash was sent to the Centers for Disease Control, where vaccine-type varicella strain was detected. This case is the least serious of the 10 adverse events because there was no symptomatic viremia after the vaccination; the case will not be further discussed in the following sections. Similar cases of herpes zoster have also occurred in immunocompetent children in the years following administration of live varicella vaccine (7). In the following sections, we will draw hypotheses about mechanisms supporting viral dissemination after live zoster vaccination.

## DISSEMINATED VIRAL INFECTION BECAUSE OF DIMINISHED INNATE AND ADAPTIVE IMMUNITY

The most common cause of death occurred in subjects who developed a persistent viremia with organ failure after live zoster vaccination. Viremia is a common event after both wild-type and vaccine-type VZV infection. Nearly all children with wild-type varicella have a viremia prior to the onset of the typical vesicular exanthem (16). Since the varicella vaccine is a live attenuated virus, there is local replication in the skin with an associated viremia in approximately 50% of the vaccinated children (17). Viremia occurs in about 50% of elderly adults with herpes zoster (18). Likewise, since the live zoster vaccine virus also replicates at the site of injection, in spite of prior immunity dating back to childhood varicella, there is an associated viremia, again in approximately 50% of the vaccinated adults (19). This viremia post-vaccination in the presence of a likely childhood varicella infection undoubtedly led to the dissemination seen in our patients following their live zoster vaccination, most likely due to mild to moderate immunosuppression in the recipients (Table 1).

Based on the data in Table 1, we propose that chronic treatment with corticosteroids coincident with administration of vaccine is a risk factor in some of these 10 cases (20). As demonstrated in early studies by Fauci, even small corticosteroid dosages can lead to transient lymphopenia (21). In turn, transient lymphopenia would lead to transient immunosuppression. The dosages in the these cases were moderate (40 mg/day) to low (5–10 mg/day). One publication about administration of live zoster vaccine to patients on prednisone (60 years and older) includes data from a clinical trial containing two test groups: Stratum I receiving 5–10 mg daily of prednisone (182 subjects) and Stratum II (25 subjects) receiving >10–20 mg daily of prednisone (22). No immunized subject developed localized or disseminated vaccine virus infection. Another publication from the United Kingdom advised physicians to use caution when administering live zoster vaccine to elderly patients who may be mildly immunocompromised (23). In the cases described in Table 1, the adverse effects of corticosteroids may have been amplified by co-administration of other immunosuppressive medications.

## DISSEMINATED VIRAL INFECTION IN PATIENTS WITH CHRONIC LYMPHOCYTIC LEUKEMIA

The fact that two of the six deaths following dissemination of zoster vaccine virus occurred in patients with chronic lymphocytic leukemia was unexpected (Table 1). Chronic lymphocytic leukemia is the cause of only 1% of all new cancers annually in the USA and the UK. When the literature was reviewed, one group of oncologists simply advised that patients with chronic lymphocytic leukemia were at intermediate risk of developing herpes zoster, even patients who had not received chemotherapy in the past months (24). These two cases suggest a reduction in as yet undefined innate immune factors that impair viral growth in patients with chronic lymphatic leukemia.

## DISSEMINATED VARICELLA INFECTION IN AN IMMUNOLOGICALLY NAÏVE PATIENT

Patient 9 undoubtedly had a primary varicella infection following his live zoster vaccination. In other words, he escaped exposure to varicella during his childhood. Unlike the others who died, this patient was likely immunocompetent. Nevertheless, primary varicella infection in an elderly person is a serious adverse event because of the risk of varicella pneumonitis, which is frequently life-threatening (1, 2). Since rash is not usually seen in live zoster vaccine recipients who have had childhood varicella, this one case illustrates that innate and adaptive immunity in other individuals with a history of childhood varicella can suppress replication of an infectious dosage of 19,000 units. However, 50% will still have a viremia in the absence of an exanthem.

## ACUTE RETINAL NECROSIS FOLLOWING LIVE ZOSTER VACCINATION

The serious adverse event called acute retinal necrosis following administration of the live zoster vaccine is so rare that we decided to compare this one case (case 8 described earlier) with two known cases of acute retinal necrosis following administration of the live varicella vaccine. The first reported case after varicella vaccine occurred in a 20-year-old man with inflammatory bowel disease and protein-losing enteropathy (25). He developed blurred vision 4 weeks after one dose of the vaccine. Ophthalmic examination identified left retinal necrosis. Virus isolated from vitreous fluid was identified as a vaccine strain by Merck & Company. The other reported case occurred in a 42-year-old VZV-seronegative man who was vaccinated with the live varicella vaccine because of an exposure to varicella at work (26). Seven weeks after vaccination, he complained of blurred vision and was diagnosed with bilateral acute retinal necrosis. Virus from the vitreous fluid and cerebrospinal fluid was identified as vaccine strain by the Centers for Disease Control, Atlanta. Extensive diagnostic screening also revealed that the man had a previously undiagnosed human immunodeficiency virus infection.

**Fig 1:**
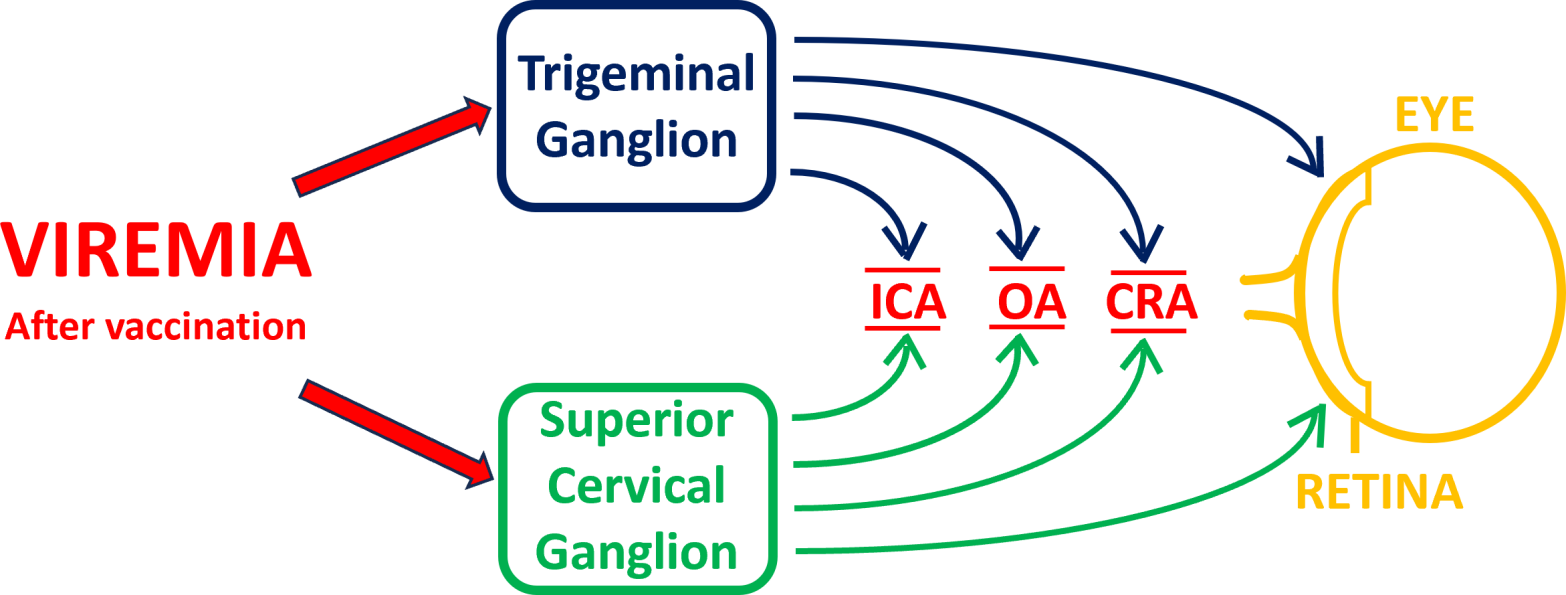
Schema for pathogenesis of acute retinal necrosis following live zoster virus vaccination. All neuronal pathways are described in cited articles in the text. ICA, internal carotid artery; OA, ophthalmic artery; CRA, central retinal artery.

The route by which VZV travels to the retina has not been clearly delineated and may not always be the same route in every patient with acute retinal necrosis. Investigations of acute retinal necrosis caused by wild-type virus have shown that acute retinal necrosis usually occurs in adults and not in children. Since wild-type virus has been isolated from enucleated diseased eyes, the disease appears to be caused by the acute infection of the retina and is not related to an immune response to viral antigen (27). Similarly, we have tabulated the serious adverse events following varicella vaccination and have not located a single reported case of isolated acute retinal necrosis caused by the vaccine virus in children aged 18 years or younger (7). The blood supply to the retina is the central retinal artery, which is a branch of the ophthalmic artery, which in turn is a branch of the internal carotid artery (28). The ophthalmic artery is innervated by afferent fibers from both the trigeminal ganglion and the superior cervical ganglion (29, 30). Therefore, a viremia following live zoster vaccination could allow the virus to enter the retina directly via the central retinal artery (Fig. 1). Alternatively, the viremia could seed either the trigeminal ganglion or the superior cervical ganglion, after which the virus could travel by afferent fibers to the ophthalmic artery (Fig. 1).

## INBORN ERRORS IN INNATE IMMUNITY

The increased susceptibility of children with inborn errors of innate immunity to recurrent infections has received considerable attention in the medical literature. The fact that a similar error in innate immunity has been identified in patients with wild-type varicella neurologic disease is less well-known. In whole-exome sequencing studies performed in Scandinavia by the Mogensen Laboratory, mutations in the RNA polymerase III subunit genes were identified in some children and adults with severe primary and severe reactivated varicella infections (31). In a more recent publication, the same group identified a boy with complicated varicella who had a polymorphism in the cyclic GMP-AMP synthase-stimulator of interferon genes (cGAS-STING) pathway (32). The authors of the 10 case reports described in Table 1 did not mention testing for these mutations. However, in the publication of serious neurologic adverse events following live varicella vaccination of children, one patient was tested and did not have an RNA polymerase III mutation (33).

## INCREASED VIRULENCE BECAUSE OF WILD-TYPE ALLELES IN THE VACCINE GENOME

All of the earlier sections mention defects in innate and adaptive immunity. In this section, we identify an explanation for increased virulence of the vaccine. The live attenuated varicella vaccine known as the Oka vaccine was produced in a traditional approach by repeated passage in both human and guinea pig cells (34). The VZV Oka DNA genome contains almost 125,000 base pairs, arranged into at least 71 open reading frames (ORFs) (35). The final live vaccine product was never cloned, for example, by limiting dilutions. Therefore, the current commercial live vaccines derived from the Oka vaccine stock virus in the Takahashi Laboratory in Osaka contain hundreds, if not thousands, of variants or subspecies (36, 37). The major differences between the varicella vaccine genome and its wild-type parental genome have been defined by several research groups in several publications (35–39). Among the 137 polymorphisms in the 71 ORFs, the most polymorphisms have been found in ORF62, a major regulatory gene product and transcription factor, also called immediate early 62 (IE62); IE62 is the homolog of the herpes simplex virus ICP4 protein. However, important analyses have shown that single nucleotide polymorphisms (SNPs) within IE62 cannot account entirely for the attenuation of the Oka vaccine strain (38).

**Fig 2:**
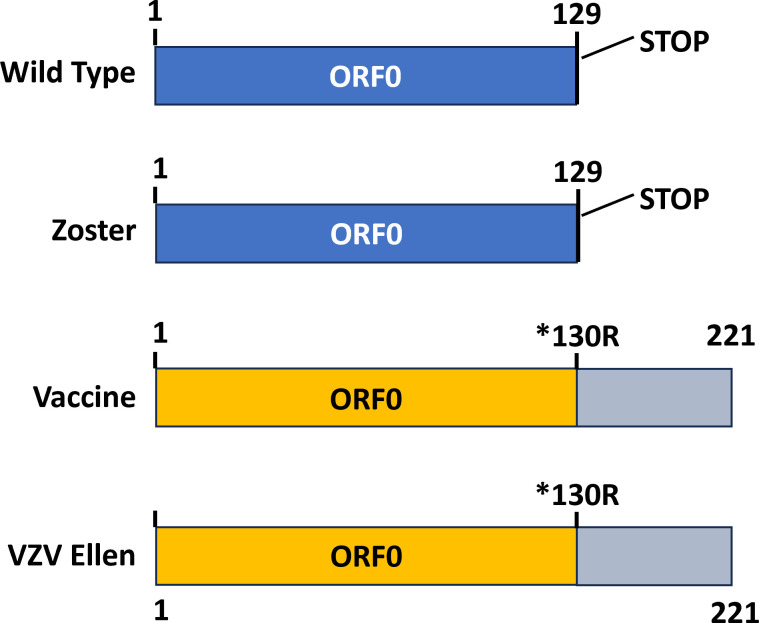
Diagram of VZV ORF0 in four varicella strains. ORF0 is the initial ORF in the unique long region of the wild-type varicella genome. The ORF0 product is a glycosylated type 2 transmembrane protein; it is the homolog of herpes simplex virus UL56. The stop codon is mutated in the vaccine strain. Wild type; Zoster, child with zoster caused by a variant vaccine genotype with wild-type ORF0 sequence. Vaccine type; Ellen, laboratory VZV strain characterized as having a vaccine-type ORF0 sequence. GenBank: wild type, AB097933; vaccine type, AB097932; VZV Ellen, JQ972913.

In this Gem article, we cannot survey all additional determinants of attenuation. We will concentrate on a determinant found over a 14-year interval in the widely used laboratory strain called VZV Ellen (39). The best available animal model to define attenuation of VZV spread in human skin is the severe combined immunodeficient (SCID) mouse model developed in the Arvin Laboratory (40). In this model, human skin sections are implanted subcutaneously within the SCID mouse and subsequently injected with VZV strains. The spread of Oka vaccine virus is remarkably slower than the wild-type virus in the human skin xenografts. For example, the mean titer in six skin specimens infected with vaccine Oka was 1,600 plaque-forming units, whereas the mean titer was 6,200 units in six implants infected with parental Oka virus. The difference was statistically significant (*P* = 0.002). In addition to decreased titers of intracellular virus, vaccine virus did not replicate at all in two of six skin implants. The laboratory strain VZV Ellen had been included in the experiment as a control virus in one experiment. Surprisingly, VZV Ellen was even more attenuated than the vaccine virus, only growing in one implant with a titer so low as to preclude further titrations. Fourteen years later, when we sequenced the Ellen genome by next-generation technology, we detected a stop codon mutation in Ellen ORF0 that was identical to the stop codon mutation in the vaccine virus (*130R) (39). The probability that two strains both attenuated in skin xenografts in the SCID-hu mouse model would acquire the same mutation by chance was highly unlikely (1 × 10E−08). In short, we hypothesized that the T560C mutation in the ORF0 stop codon did not occur by chance; instead, this mutation (*130R) was likely to be a major determinant of vaccine attenuation (Fig. 2). A later comprehensive bioinformatics analysis of the 137 vaccine SNPs came to the same conclusion that the ORF0 SNP (*130R) as well as two IE62 SNPs (nucleotides 106262, 107252) were the most likely determinants of attenuation (36).

By serendipity, 7 years later, we cared for a 3-year-old boy with severe herpes zoster in the left lumbosacral region (41). The discomfort was so great that the boy refused to walk and was carried into the emergency department by his father. The boy had been immunized at age 20 months. Because of the severity of the herpes zoster, we speculated that the boy may have had an unrecognized breakthrough infection with wild-type virus. When we performed partial sequencing on the virus recovered from a vesicle in the skin rash, however, we did not detect a wild-type virus. Instead, the genome had expected fixed alleles in IE62 found in the vaccine strain, but more importantly lacked the ORF0 stop codon mutation usually found in the Oka vaccine strain (Fig. 2). Since extensive sequencing of wild-type strains has failed to find evidence of recombination between wild-type varicella viruses and vaccine-type varicella viruses, we postulate that the isolate recovered from the child with severe herpes zoster was a more virulent variant of the vaccine strain that reactivated in the young boy about 1 year after his first varicella vaccination. By variant, we simply mean an isolate whose genome sequence differs from that of a reference virus with its GenBank sequence.

The Breuer Laboratory has shown by deep sequencing of several Oka vaccine vials that an average of 5%–7% of the infectious units within each vial of live varicella vaccine or live zoster vaccine are genotypes that harbor the wild-type ORF0 allele rather than the vaccine-type ORF0 allele (Fig. 2) (36). Thus, the viremia accompanying a zoster episode following vaccination could include variants with the wild-type ORF0 mutation. In short, more virulent variants of the vaccine virus may be a contributing factor to the fatal outcomes in the six cases of viral dissemination described in this article. None of the viral isolates from the blood from the six fatal cases were subjected to deep sequencing, as per the data in the case reports.

## CONCLUSION

There is no animal model with an intact immune system that replicates the entire VZV infectious cycle with its viremic phase (42). Therefore, we have analyzed severe adverse events following live vaccination of human subjects, in order to gain insight into VZV pathogenesis. Authors of these 10 reports generally attributed the adverse event to inadvertent immunization of an immunocompromised host. After conducting this survey according to guidelines provided to authors of Gem articles, it is our opinion that two other mechanisms should be considered. First, we propose that the viremia in a fatal dissemination case may include a minor vaccine genotype that is more virulent than the more common vaccine genotype with an intact core of attenuating single nucleotide polymorphisms (35–39). Based on this model, we propose that some deaths following live dissemination were caused not only by impaired immune responses but also a viremia in which one of the genotypes was a vaccine variant, such as those with a wild-type ORF0 allele (Fig. 2). We did not find any report that analyzed by deep sequencing viral genomes recovered from a viremia following zoster vaccination.

Second, we propose that acute retinal necroses after vaccination may have a very odd contributory mechanism, namely, which extremity is injected. We found three reported cases of acute retinal necroses, one after live zoster vaccination and two after live varicella vaccination. What is odd is that the two cases of retinitis following varicella vaccination occurred in older individuals (age: 20 and 42 years) who likely had a vaccination in the upper arm. We found no reported case of acute retinal necrosis caused by varicella vaccine in children immunized in the first few years of life, where the injection is routinely given in the thigh, even though hundreds of million doses have been administered (7). The live zoster vaccination is routinely administered in the upper arm. The simplest explanation is that retinitis requires a proximal viremia following vaccination nearer the head for the virus to find its way to the trigeminal or superior cervical ganglia (Fig. 1). Injection in the thigh would lead to a lower likelihood of virus to reach its target ganglion. Other less important differences in responses by humans to vaccination can be dependent on the extremity in which the vaccine is injected. For example, there are differences in immunogenicity after vaccination between ipsilateral and contralateral arms and differences in reactogenicity after vaccination of an arm or a leg (43, 44). An obvious limitation is that we cannot account for the adverse events that are never reported.

## Data Availability

All data published in this article are contained within the cited references.
